# Vascular Connections Into the Grape Berry: The Link of Structural Investment to Seededness

**DOI:** 10.3389/fpls.2021.662433

**Published:** 2021-04-15

**Authors:** Zeyu Xiao, Sabrina Chin, Rosemary G. White, Aude M. Gourieroux, Vinay Pagay, Stephen D. Tyerman, Leigh M. Schmidtke, Suzy Y. Rogiers

**Affiliations:** ^1^Australian Research Council Training Centre for Innovative Wine Production, Adelaide, SA, Australia; ^2^National Wine and Grape Industry Centre, Charles Sturt University, Wagga Wagga, NSW, Australia; ^3^Noble Research Institute LLC, Ardmore, OK, United States; ^4^Commonwealth Scientific and Industrial Research Organisation (CSIRO) Agriculture and Food, Acton, ACT, Australia; ^5^Department of Wine Science, The University of Adelaide, Glen Osmond, SA, Australia; ^6^New South Wales (NSW) Department of Primary Industries, Wagga Wagga, NSW, Australia

**Keywords:** berry anatomy, vascular tissues, xylem, phloem, imaging, *Vitis vinifera*

## Abstract

Vascular bundles in the grape pedicel and berry contain the conduits, phloem and xylem, for transport of water, sugar, nutrients and signals into and through the grape berry and play a critical role in berry growth and composition. Here, we assess the vascular anatomy within the proximal region of the berry. Guided using a 3D berry model generated by micro-CT, differential staining of transverse sections of berries and receptacles was followed by fluorescent microscopy. Morphometric and vascular characteristics were analyzed within the central proximal region (brush zone, a fibrous extension from the pedicel vascular system into the berry) of the seeded cultivars Shiraz and Sauvignon Blanc, as well as the stenospermocarpic cultivars Ruby Seedless and Flame Seedless. Observations revealed a change in vascular arrangement from the receptacle into the berry brush zone and differences in xylem element size as well as xylem and phloem area relationships. Xylem anatomical and derived hydraulic parameters, as well as total tissue area of xylem and phloem varied between cultivars and in receptacle and berry components. Variation in vascular growth between grape pedicels and berries was independent of seededness. Differences in receptacle xylem vessel size and distribution could contribute to cultivar-dependent xylem backflow constraint.

## Introduction

Growth of fleshy fruits such as the grape (*Vitis vinifera*) depends on the coordination between vascular transport and cell expansion within the fruit (Matthews and Shackel, 2005). Grapevine vascular bundles transport water, nutrients and photo-assimilates (Rogiers et al., 2001, 2006b; Tilbrook and Tyerman, 2009; Keller et al., 2015; Knipfer et al., 2015) and therefore play a critical role in berry growth and composition. The balance of water influx and efflux dictates both yield and fruit quality (Coombe, 1992). In addition, plants translocate developmental and defense molecules through the vascular system (Williams et al., 2002; Friml, 2003). Vascular bundles are composed of xylem, phloem and meristematic tissues (vascular cambium) (Sachs, 1981). Differentiation from the cambium results in addition of xylem and phloem both inwards and outwards (Jouannet et al., 2015). The differentiation and growth of the vascular system are hormonally controlled with both environmental and genetic cues that regulate cambium activity and formation (Aloni, 1987; Scarpella and Meijer, 2004).

The xylem is the main conduit for water and minerals from roots to the shoot, while phloem transports photo-assimilates from the source tissues (leaves) to the sinks (such as the fruit) (Scarpella and Meijer, 2004). The water economy of the grape berry, and more specifically the contribution of the two vascular systems, has been extensively investigated in recent decades. During the pre-veraison stages of grape berry development, xylem plays a key role in supplying water to the fruit to ensure cell and berry expansion. However, the relative contribution of the xylem vs. the phloem to the berry’s water supply changes at veraison (During et al., 1987; Findlay et al., 1987; Greenspan et al., 1994, 1996; Keller et al., 2015). With the onset of ripening, flow into the grape through the xylem gradually declines even though pedicel xylem vessels through the receptacle/berry juncture remain connected (Rogiers et al., 2001; Tyerman et al., 2004; Choat et al., 2009; Knipfer et al., 2015; Scharwies and Tyerman, 2017). Peripheral xylem strands located just beneath the skin in the outer pericarp also remain intact (Chatelet et al., 2008b) with stretching and continuous differentiation of the tracheary elements after veraison to accommodate rapid berry expansion (Chatelet et al., 2008a). Phloem becomes the main conduit supplying water for berry growth and transpiration post-veraison, with a transition from symplastic to apoplastic unloading of sugar within berries of the Kyoho grape cultivar (Zhang et al., 2006). It has been suggested that during ripening, xylem may facilitate the recycling of phloem-derived water (water backflow) (Zhang and Keller, 2017) and the cultivar-dependent variation in xylem hydraulic connection could result in differences in pre-harvest berry weight loss amongst cultivars (Tilbrook and Tyerman, 2009; Scharwies and Tyerman, 2017).

The receptacle/berry junction is a critical branching point for vascular tissues that supply either the peripheral network under the berry skin or the central bundles traversing the core of the berry. These central bundles branch off to form the ovular bundles that supply the placenta/seeds, or they continue through the berry interior to the stylar remnant at the berry apex (Harris et al., 1968; Pratt, 1971). The central region of the berry immediately distal to the receptacle is referred to as the brush zone (Figure 3A). The brush zone is composed of vascular tissue and associated parenchyma cells and is the tissue that remains behind when a ripe berry is plucked from the pedicel (Mullins et al., 1992). Grape seeds have a high respiratory demand prior to lignification of the testa (Xiao et al., 2018b) and are strong mineral sinks (Rogiers et al., 2006a). Maximum fresh seed weight occurs around veraison while dry weight accumulation typically continues at a slow rate for a few more weeks (Ristic and Iland, 2005). Unlike the seeded wine grape cultivars, seedless cultivars are popular for the table grape and dried fruit industries. The seedlessness in these cultivars, such as Ruby Seedless and Flame Seedless, is typically the result of stenospermocarpy (Gribaudo et al., 1993; Akkurt et al., 2019), in which the embryo is aborted shortly after fertilization leaving only small seed remnants (Pratt, 1971).

The anatomy of the central vasculature in the grape berry has not yet been well-described, perhaps due to the challenging location of this tissue. Embedded within several soft layers of mesocarp cells, it is difficult to section and observe. Here we aimed to assess the vascular anatomy within the proximal region of the berry, specifically from the receptacle into the brush zone, prior to and after veraison in seeded and seedless cultivars, using natural fluorophore (lignin) and two fluorescent stains, acridine orange and aniline blue fluorochrome, as well as X-ray micro-computed tomography (micro-CT) to aid in tissue identification.

## Materials and Methods

### Plant Materials

Grape berries (*Vitis vinifera*) were sampled in two growing seasons, 2017/2018 and 2018/2019, at the National Wine and Grape Industry Centre experimental vineyard, Wagga Wagga, Australia (35.1583S, 147.4573E, elevation: 212 m). The vineyard was planted in 2003 on a sandy-loam over very hard medium clay. Own-rooted vines were spaced at 1.5 m intervals in rows 3 m apart, spur pruned (2 buds per spur) with 20 buds per vine and trained to a bilateral cordon. The block consisted of six rows including the five genetically distinct cultivars in a randomized complete block design with each cultivar in a 3-vine panel. The vineyard floor was composed of voluntary species that were slashed regularly during spring. The vines were drip irrigated (4 ML/ha over the season) and managed according to recommended best practice for the Australian wine industry.

In the 2017/2018 season, 50 berries of Shiraz (clone BVRC12 and 1654), Sauvignon Blanc, Ruby Seedless and Flame Seedless were collected weekly between 44 and 132 days after anthesis (DAA) (around 14/11/2017). A sub-sample of 30 berries was then fixed in 100% methanol and stored at 4°C. In 2018/2019, 30 berries each cultivar of the same cultivars were collected at 65 DAA (around 5/11/2018).

### Berry Growth Measurements

Phenological stages were assessed according to the E-L system (Dry and Coombe, 2004). Berry total soluble solids (TSS, °Brix) and berry fresh weight (g) were measured weekly, using a sub-sample of 5 and 10 berries, respectively, during the 2017/2018 growing season. TSS was measured using a digital refractometer (Atago, Tokyo, Japan). In the 2018/2019 season, berry fresh weight (a sub-sample of three berries) and TSS (a sub-sample of four berries) were also recorded.

### X-Ray Micro-Computed Tomography (Micro-CT)

A Quantum GX (PerkinElmer, Waltham, Massachusetts, United States) was used to scan a Shiraz grape berry soon after veraison. A total of 803 two-dimensional projections were acquired with 0.448° angular steps, at 90 kV/88μA. Total scanning time was 14 min. Voxel size was approximately 72 μm. Quantum GX (PerkinElmer, Waltham, Massachusetts, United States) was used for slice reconstructions. The 3D berry model was built in Amira 3.6 (Thermo Fisher Scientific, Bordeaux, France) using the 3D volume rendering module then the clipping Plane module was used to generate a 2D plane for visualizing the berry interior with minimal sample handling and image manipulation.

### Berry Sectioning and Staining

Methanol-fixed berries at 44 and 85 DAA from the 2017/2018 season were sub-sampled (*n* = 3 or 4) and hand sectioned transversely at the receptacle, the receptacle/berry junction (0–2 mm from receptacle/berry junction) and brush zone (2–4 mm distal from the junction). Sections were incubated in acridine orange (0.01% w/v in H_2_O, Sigma-Aldrich) at room temperature for a minimum of 15 min followed by two 5-min washes with water. At least three berries for each cultivar were sectioned, stained and imaged. Because phloem tissues are thin and can be difficult to differentiate from neighboring tissue, aniline blue fluorochrome was used to stain callose, as it accumulates immediately on the sieve plates upon severing the phloem (Sjolund, 1997; Stass and Horst, 2009). In the 2018/2019 season, sub-sampled fresh berries (*n* = 3 or 4) were hand sectioned transversely at the same positions as for the methanol-fixed berries and at an additional position at brush zone 2 (4–6 mm distal from the junction). These sections were incubated in aniline blue fluorochrome solution (0.025% w/v in H_2_O, Biosupplies Australia) for a minimum of 30 min at room temperature followed by two 5-min washes with water.

### Microscopy Imaging

Fluorescence images of the stained sections were collected using an Olympus Provis AX70 (Olympus Optical, Tokyo, Japan) microscope, using two fluorescence filters combinations (excitation filter 330–385 nm, dichroic mirror 400 nm, barrier filter 420 nm, for methanol-fixed and fresh berries and excitation filter 520–550 nm, dichroic mirror 565 nm, barrier filter 580 nm for methanol-fixed berries only), with an Olympus DP80 digital camera.

### Xylem and Phloem Anatomy

Using hand-drawn ellipses in ImageJ (Schneider et al., 2012), semi-major (*a*_1_, μm) (half of Feret diameter) and semi-minor (*b*_1_, μm) (half of minimum-Feret) axes were determined for xylem vessels in the receptacle, receptacle/berry junction and brush zone (*n* = 3 or 4, 15–70 vessels were randomly selected in each image, Supplementary Figure 1), in both seasons and all cultivars. Idealized vessel diameter (*d*_1_, μm), as the equivalent circle diameter for the lumen was obtained using Eq. (1) (White, 1991),

(1) d1=(32×(a1×b1)3(a12+b12))14

The idealized diameters were used to calculated the mean hydraulically weighted diameter (*d*_*h1*_, μm) according to Eq. (2) (Sperry et al., 1994),

(2) dh⁢1=∑d15∑d14

To estimate the frequency distribution of receptacle xylem vessels size classes and their potential contribution to the area-specific hydraulic conductivity (*K*, kg m^–1^ MPa^–1^ s^–1^), xylem region(s) were selected in the cross-sectional images (all cultivars in season 2018–2019) using ImageJ. Semi-automated segmentation was applied, yielding 198–276 vessels (Supplementary Figure 2) per image, to measure, single and total vessel lumen area (*A*_lumen_, μm^2^), vessel number (*n*) and density (VD, *n* mm^–2^), semi-major (*a*_2_) and semi-minor (*b*_2_) axes (μm) of individual vessels in the corresponding xylem area (*A_*c*__–__xylem_*, μm^2^). The idealized vessel diameter *d*_2_ (μm) of individual vessels and subsequently the mean hydraulically weighted diameter (*d*_h2_, μm) were calculated as previously defined. Relative lumen area (*A*_lumen_, %) was obtained by dividing total *A*_lumen_ (μm^2^) by the xylem area (*A_*c*__–__*xylem*_*, μm^2^). Assuming the selected xylem region(s) used for the image segmentation was representative for each cross-sectional xylem, the potential area-specific hydraulic conductivity (*K*, kg m^–1^ MPa^–1^ s^–1^) of the receptacle, for vessels in each size class (i.e., 4 μm class interval in *d*_2_) were estimated according to the Hagen-Poiseuille equation, Eq. (3) (Tyree and Ewers, 1991),

(3) K=(π⁢ρ128⁢η⁢Ac-x⁢y⁢l⁢e⁢m)⁢∑(d24)

where *η* is the viscosity of water (1.002 × 10^–9^ MPa s), *ρ* the density of water (998.2 kg m^–3^), both at 20°C. The percentage of *K* of each vessel size class contributed to the total *K* was the determined.

The tissue areas of xylem (*A*_*xylem*_) and phloem (*A*_phloem_) in the berry receptacle and brush zone cross-sections were estimated using the area measurement function in ImageJ on hand traced area outlines for all cultivars in both seasons (*n* = 3 or 4). In the cases where only part of a large receptacle could be imaged at the required magnification, tissue areas in an approximate quadrant of the receptacle were measured and multiplied by 4 to estimate the total respective tissue areas in the transverse section (Supplementary Figure 3).

### Statistical Analysis

Sugar accumulation curves during the 2017/2018 season were fitted with a logistic growth function (Eq. 4) and compared across the five cultivars using the Akaike information criterion.

(4) $$Y=\frac{Y_{M}\times Y_{0}}{\left(Y_{M}-Y_{0}\right)\times e^{\left(-k\times x\right)}+Y_{0}}$$

where *Y*_0_ represents the initial level of TSS following the lag-phase constrained to 0–5°Brix, *Y*_*M*_ is the maximum TSS constrained to 32°Brix and k is the rate constant (day^–1^).

For the 2018/2019 season, berry weight, TSS, VD, *A*_lumen_ (%) and *K* across the cultivars were compared using a one-way ANOVA followed by Fisher’s LSD tests. Xylem vessel abundance in five diameter classes and their respective contribution to hydraulic conductivity (*K*) were compared using two-way ANOVA, followed with Fisher’s LSD tests. For both seasons, *A*_*xylem*_ and *A*_phloem_ were compared between and within cultivars using two-way ANOVA, testing for cultivar (C), section position (SP) and cultivar × section position (C × SP) effects, followed with Fisher’s LSD tests, where C and/or SP effects were significant. Relationships between phloem and xylem tissue areas in 2017/2018, before and after veraison, across different cultivars were analyzed using parametric correlations (Pearson’s *r*). All analyses were carried out using Prism 8 (GraphPad Software, San Diego, California, United States).

## Results

### Berry Ripening

**FIGURE 1:**
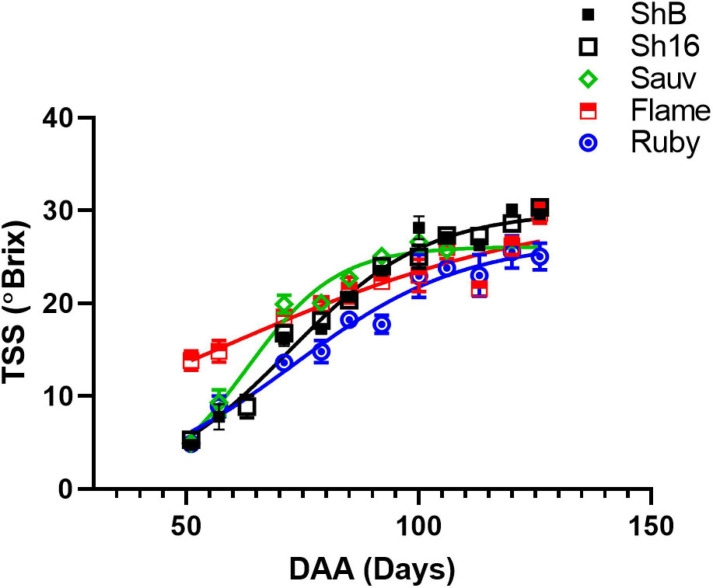
Sugar accumulation of Shiraz BVRC12 (ShB) and 1654 (Sh16), Sauvignon Blanc (Sauv), Flame Seedless (Flame), and Ruby Seedless (Ruby) during season 2017/2018. Total soluble solids (TSS) were measured and are shown as means ± s.e.m. (*n* = 5). Where separate lines are shown this indicates that there was a significant difference between the fitted lines (logistic growth, Akaike information criterion).

**FIGURE 2:**
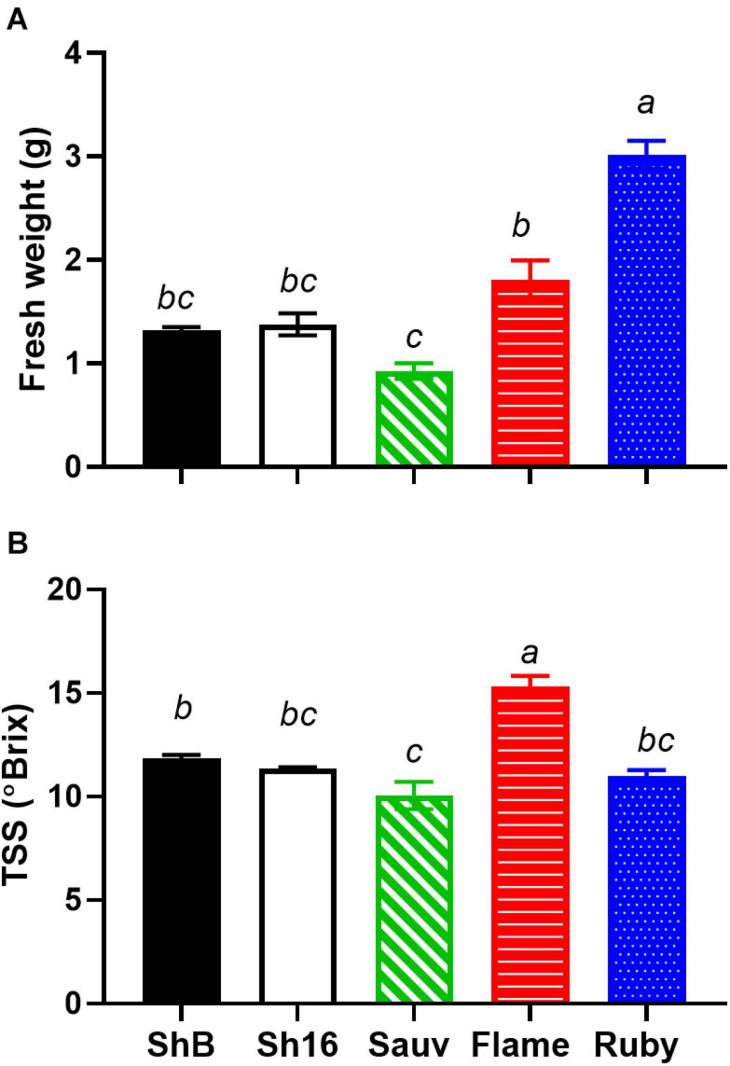
Fresh weight **(A)** and total soluble solids (TSS) **(B)** of Shiraz BVRC12 (ShB) and 1654 (Sh16), Sauvignon Blanc (Sauv), Flame Seedless (Flame), and Ruby Seedless (Ruby) at around 65 DAA in season 2018/2019 [*n* = 3 in **(A)**, *n* = 4 in **(B)**, mean ± s.e.m.]. Different lower-case letters indicate statistical differences (One-way ANOVA, Tukey’s test, *P* < 0.05).

The vasculature is the nutrient, water and carbohydrate conduit into the berry and important for sugar ripeness, and might be expected to develop in parallel with berry ripening. Sugar accumulation into berries of the 2017/2018 season is shown in Figure 1. At the first sampling time, approx. 51 DAA, Flame Seedless berries had considerably higher total soluble solids (TSS) (13.84 ± 1.05°Brix) than the other cultivars (mean of all others = 4.95 ± 0.11°Brix). At the last sampling, approx. 126 DAA, Ruby Seedless had lower TSS (25.06 ± 1.44°C) compared to Flame Seedless (29.70 ± 1.10°C) and the two Shiraz clones (BVRC: 29.60 ± 0.45°Brix, 1654: 30 ± 0.56°Brix). The sampling for the anatomical study at 44 and 85 DAA thus occurred during the pre-veraison and post-veraison (approx. 21°Brix) stages, respectively. As a function of time, TSS was best fitted by Eq. (4) and different curves were best-fitted to each cultivar except one curve can describe the two Shiraz clones (Figure 1). Noticeably, the rate constants were 0.03 day^–1^ for Flame Seedless, 0.05 day^–1^ for Ruby Seedless, 0.07 day^–1^ for both Shiraz BVRC12 and 1654 and 0.1 day^–1^ for Sauvignon Blanc. Ruby Seedless berries weighed approximately twice as much as the berries of Sauvignon Blanc and the two Shiraz clones from 71 to 132 DAA (Supplementary Figure 4). In the 2018/2019 season, at approximately 65 DAA, the two seedless cultivars had berries with larger mass (1.81 ± 0.18 g for Flame Seedless and 3.02 ± 0.14 g for Ruby Seedless) relative to the other cultivars (Figure 2A). Berries had similar TSS (around 12°Brix) except for Flame Seedless which appeared to be more advanced in sugar ripeness (15°Brix) (Figure 2B).

### Vascular Arrangement

**FIGURE 3:**
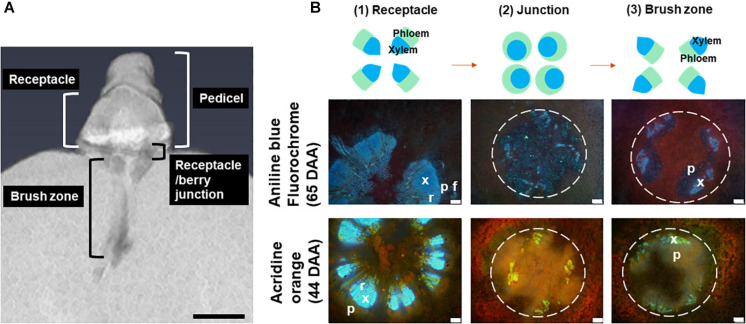
A longitudinal section of a 3D berry reconstructed using micro-CT, showing the locular cavity/vascular connection from the pedicel to the berry. Scale bar = 1 mm **(A)**. A schematic drawing of change in central vascular bundles xylem and phloem arrangement in three positions including the receptacle (1), junction (0–2 mm from receptacle/berry junction) (2) and the brush zone (2–6 mm from the junction) (3) and image examples of aniline blue fluorochrome and acridine orange stained Sauvignon Blanc (white dashed circles indicate central vascular bundles; x, xylem; p, phloem; r, xylem ray; f, phloem fiber). Excitation filter 330–385 nm, dichroic mirror 400 nm, barrier filter 420 nm. Scale bar = 100 μm **(B)**.

**FIGURE 4:**
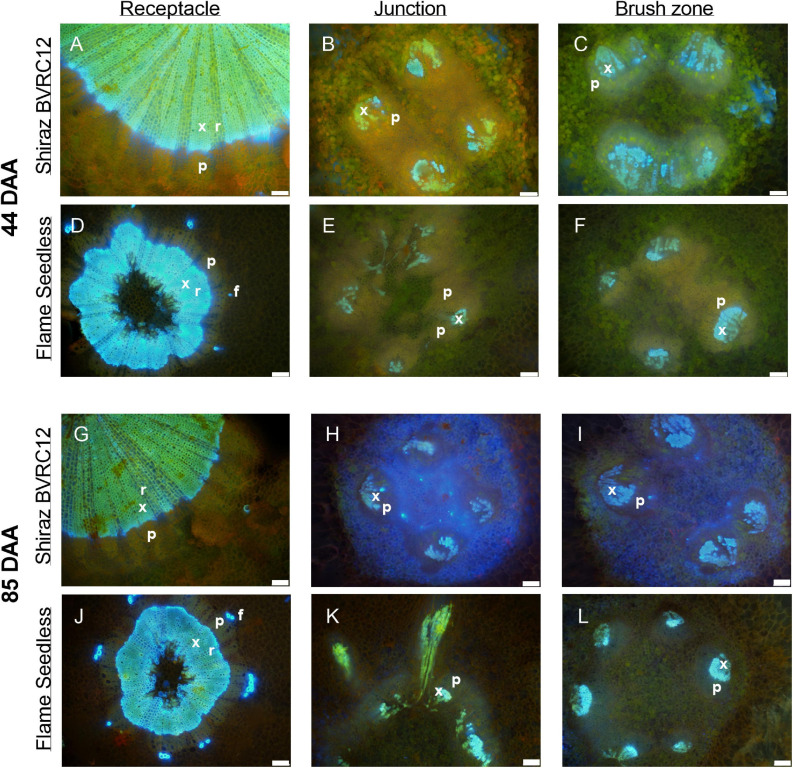
Sections of receptacles **(A,D,G,J)**, receptacle/berry junction **(B,E,H,K)**, brush zone (2–4 mm from junction) **(C,F,I,L)**, stained with acridine orange and imaged under UV, of methanol stored Shiraz BVRC12 **(A–C,G–I)** and Flame Seedless **(D–F,J–L)** at around 44 (pre-veraison) and 85 (post-veraison) days after anthesis (DAA) in the 2017/2018 season. Bright blue indicates lignin (xylem and phloem fiber), pinkish orange indicates the location of phloem (x, xylem; p, phloem; r, xylem ray; f, phloem fiber). Excitation filter 330–385 nm, dichroic mirror 400 nm, barrier filter 420 nm. Scale bar = 100 μm.

**FIGURE 5:**
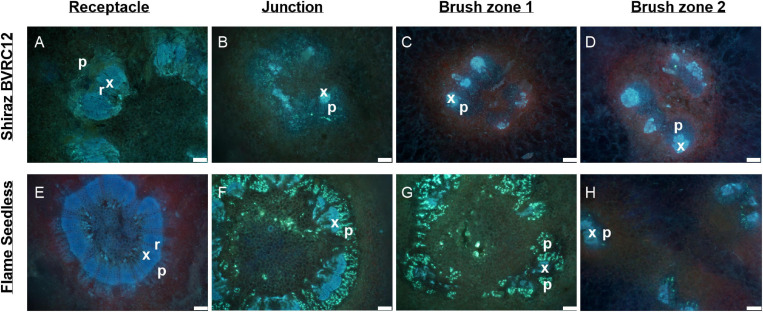
Autofluorescence of vascular bundles in the brush zone of post-veraison (85 DAA, season 2017/2018) Shiraz BVRC **(A,B)**, Sauvignon Blanc **(C,D)**, and Flame Seedless (methanol-fixed) with two filters combinations applied: (1) excitation filter 330–385 nm, dichroic mirror 400 nm and barrier filter 420 nm, showing intense blue fluorescence of xylem **(A–C)** and intense blue in the cell lumens surrounding the vascular bundles **(A)**; (2) excitation filter 520–550 nm, dichroic mirror 565 nm and barrier filter 580 nm, showing strong green fluorescence of extractives in the cell lumens sounding the vascular bundles **(B,D,F)**. Scale bar = 100 μm.

**FIGURE 6:**
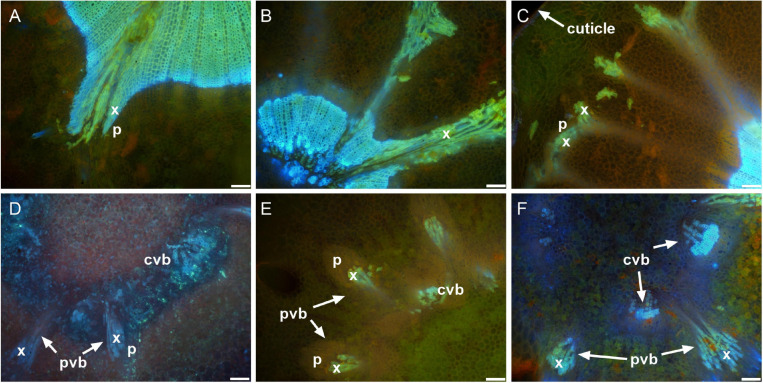
Sections of receptacles **(A,E)**, receptacle/berry junction **(B,F)**, brush zone 1 (2–4 mm from junction) **(C,G)**, brush zone 2 (4–6 mm from junction) **(D,H)**, stained with aniline blue fluorochrome and imaged under UV, of fresh berries of Shiraz BVRC12 **(A–D)** and Flame Seedless **(E–H)** at around 65 days after anthesis (post-veraison), in the 2018/2019 season. Blue color indicates lignin (xylem and phloem fiber). Bright fluorescent greenish blue indicates callose formed within the sections and primarily within phloem, red tint in images indicates chlorophyll (x, xylem; p, phloem; r, xylem ray; f, phloem fiber). Excitation filter 330–385 nm, dichroic mirror 400 nm, barrier filter 420 nm. Scale bar = 100 μm.

**FIGURE 7:**
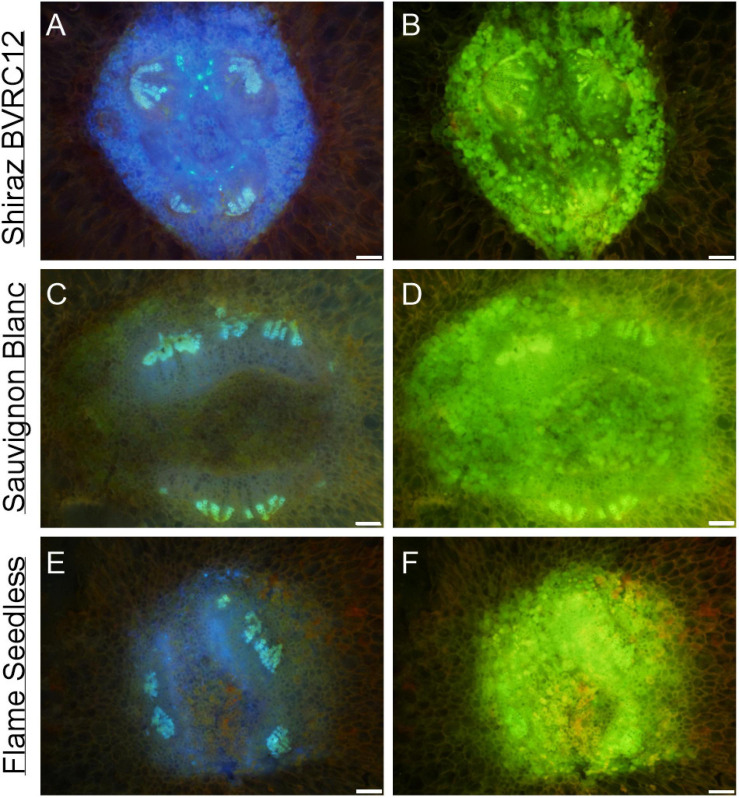
Peripheral vascular bundles in the receptacle and the receptacle/berry junction. Receptacle vascular system branched toward the epidermis of the receptacle in the pre-veraison Shiraz BVRC12 **(A)**, post-veraison Sauvignon Blanc **(B)**, and pre-veraison Flame Seedless **(C)**. Vascular bundles in the receptacle/berry junction of post-veraison Shiraz 1654 **(D)**, pre-veraison Flame Seedless **(E)**, and post-veraison Ruby Seedless **(F)** (x, xylem; p, phloem; pvb, peripheral vascular bundle; cvb, central vascular bundle). Excitation filter 330–385 nm, dichroic mirror 400 nm and barrier filter 420 nm applied to methanol-fixed sections stained with acridine orange **(A–C,E,F)** and fresh section stained with aniline blue fluorochrome **(D)**. Scale bar = 100 μm.

**FIGURE 8:**
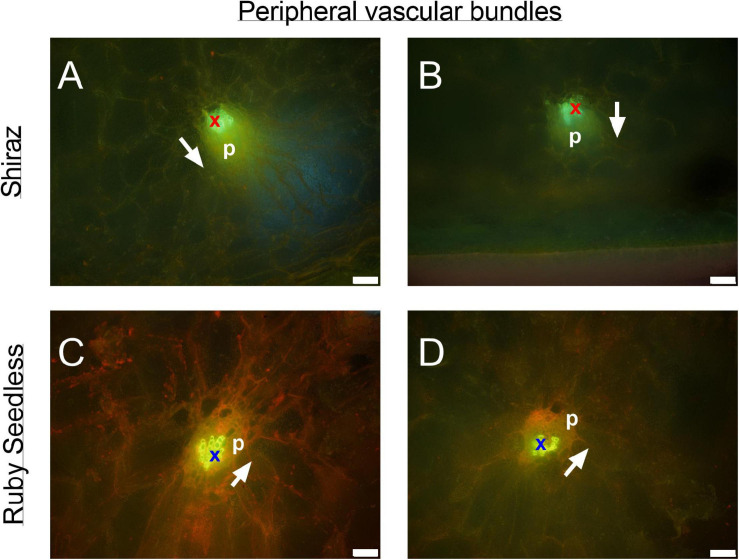
Peripheral bundles at the equator of the berries in pre-veraison (44 DAA) Ruby Seedless **(A,B)** and post-veraison (85 DAA) Shiraz **(C,D)** berries. Bright green indicates auto-fluorescent xylem vessels (x) adjacent to yellowish green phloem tissue (p) within each peripheral vascular bundle. White arrows point toward the position of the epidermis. Excitation filter 330–385 nm, dichroic mirror 400 nm, barrier filter 420 nm. Scale bar = 50 μm.

Figure 3A depicts a computational longitudinal section through a 3D berry model from a micro-CT scan to show the interior of the portion on which the dissection was made. A schematic drawing summarizes the typical change in vascular arrangement within the sectioned regions (Figure 3B). Under UV light, xylem appeared mostly bright blue due to autofluorescence (Figures 4, 5, 6) or occasionally yellowish-green following the acridine orange staining (Figures 4A,B,G, 6A–C,E, 7C, 8), whereas the phloem stained pink to orange (Figures 4A–F), or a low intensity greenish-blue (Figures 4H,I,K,L). The aniline blue fluorochrome highlighted the phloem by staining the scattered callose a bright greenish-blue (Figure 5). This was consistent across all the cultivars (Figure 5 and Supplementary Figure 6).

The transverse fluorescence microscopy images of the berry receptacle highlighted a substantial inner region of lignified secondary xylem (blue auto-fluorescence) in cell files radiating outwards from the pith (Figures 4A,D,G,J, 5A,E and Supplementary Figures 5, 6). In some receptacles, the thick-walled xylem cell files were interspersed with files of non-lignified cells (Figure 5A and Supplementary Figures 5, 6) and this was not cultivar or developmental stage dependent. At the receptacle/berry junction, the central vascular bundles were no longer arranged in one concentric ring, but rather a few distinct bundles each containing xylem and phloem tissues; this pattern was apparent in all cultivars (Figures 4B,E,H, 5B and Supplementary Figures 5, 6). Notably, some phloem surrounded each of the xylem vessels (Figures 4E,H, 5B and Supplementary Figures 5, 6). Deeper into the brush zone, phloem was oriented closer to the central axis of the grape while the xylem bundles were located on the outer edge, adjacent to the mesocarp (Figures 4C,F,I,L, 5C,D,H and Supplementary Figures 5, 6). In methanol-fixed post-veraison berries of the Shiraz clones, an intense blue fluorescence was visible in the walls of cells surrounding the central vascular bundles in the brush zone, which clearly separated them from the surrounding mesocarp parenchyma cells (Figures 4H,I, 7A and Supplementary Figure 5). The contents of the cell lumina surrounding the central vascular bundles were characterized by an intense green autofluorescence in Shiraz BVRC, Sauvignon Blanc and Flame Seedless (Figures 7B,D,F). The main vascular bundle in the receptacle was found to branch laterally, as apparent in sections close to the receptacle/berry junction (Figures 6A–C), linking to the berry peripheral vascular network base in the receptacle (Figures 6B,C). At the receptacle/berry junction, peripheral vascular bundles entered the berry as the extension of the network base in the receptacle, angled and already separated from the central vascular bundles (Figures 6D–F). The xylem of the peripheral bundles was consistently oriented toward the center of the berry while the phloem was located toward the epidermis (Figures 6E, 8).

**FIGURE 9:**
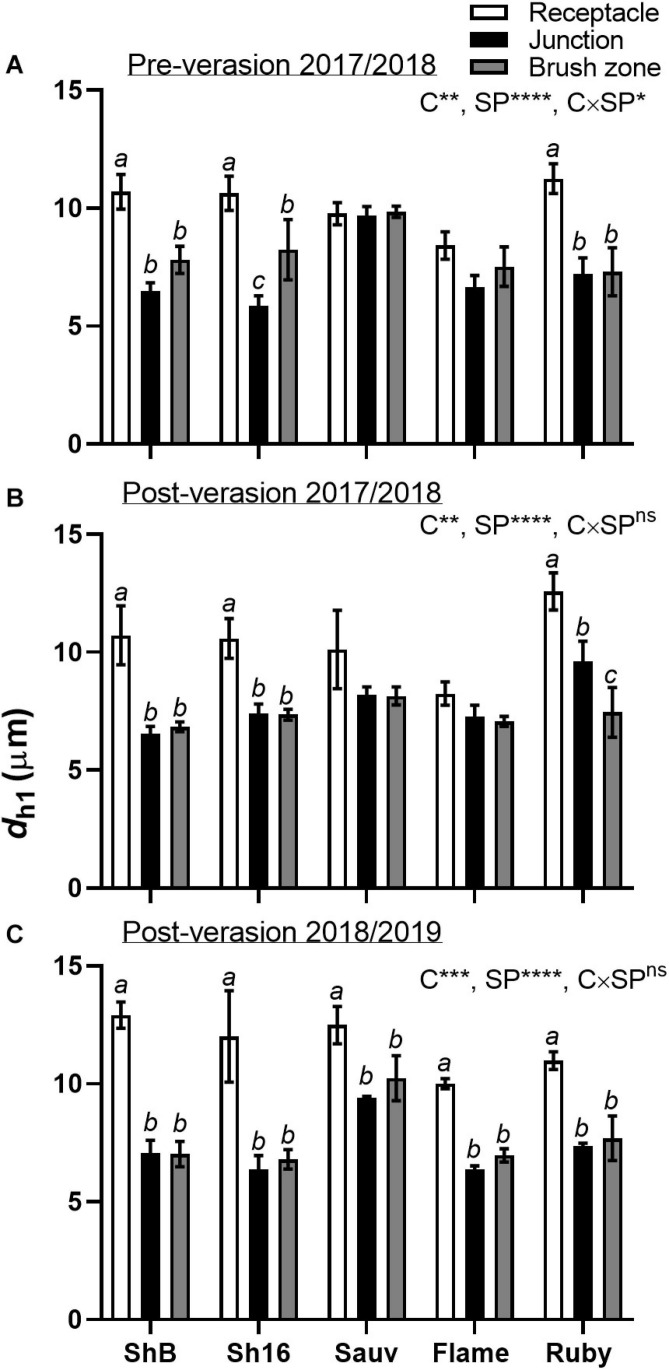
Mean hydraulically weighted diameter (*d*_*h1*_, μm) of xylem vessels in the receptacle (white bars), receptacle/berry junction (black bars) and brush zone (gray bars) of Shiraz BVRC12 (ShB), Shiraz 1654 (Sh16), Sauvignon Blanc (Sauv), Flame Seedless (Flame), and Ruby Seedless (Ruby) before **(A)** and after **(B)** veraison in the 2017/2018 season and after veraison in the 2018/2019 season **(C)** (data of brush zone 2 was used for Post-veraison 2018/2019). Data are presented as mean ± s.e.m. (*n* = 3 or 4). The ANOVA results are given for cultivar (C), section position (SP) and cultivar × section positon (C × SP) interaction. Levels of significance are *P < 0.05, **P < 0.01, ***P < 0.001, ****P < 0.0001 and *ns*, not significant. Where different lower-case letters are shown, it indicates differences between section positions within each cultivar (Fisher’s LSD test, *P* < 0.05).

Differences in the xylem mean hydraulically weighted diameter (*d*_*h1*_) were apparent for cultivar, section position and cultivar × section position (Figure 9). Calculated from hand-drawn ellipses, *d*_*h1*_ was larger in the receptacle than in the berry/receptacle junction and the brush zone in the Shiraz clones and Ruby Seedless in both seasons, and in Sauvignon Blanc and Flame Seedless after veraison in 2018/2019 (Figures 9B,C).

By analyzing all xylem lumina within the selected xylem regions in the receptacles, xylem mean hydraulically weighted diameter (*d*_*h2*_) of Shiraz BVRC12 was found to be larger than Sauvignon Blanc and the two seeded cultivars (Table 1). Vessel density (VD) in both Shiraz clones were smaller compared to the seedless cultivars (Table 1). The relative lumen area (*A*_*lume*__*n*_, %) did not differ between cultivars (Table 1). Together this indicated that a higher percentage of larger xylem vessels could exist in the Shiraz receptacles. While the total receptacle xylem area-specific hydraulic conductivity (*K*) showed no difference between cultivars (Table 1), upon further calculating the coefficient of variance (CoV) of *K*, the seeded wine grape receptacles were found to show higher variability in *K* (Shiraz BVRC12 CoV = 22.4%, Shiraz 1654 CoV = 24.9%, Sauv CoV = 25.6 %, *n* = 3) than the seedless cultivars (Flame CoV = 9.3%, Ruby CoV = 7.7%, *n* = 3 or 4).

**TABLE 1:** Receptacle xylem vessel mean hydraulically weighted diameter (*d*_h2_) (semi-automatically segmented), vessel density (VD), relative lumen area (*A*_lumen_), and total potential area-specific hydraulic conductivity (*K*) of Shiraz BVRC12, Shiraz 1654, Sauvignon Blanc, Flame Seedless, and Ruby Seedless from season 2018–2019.

Cultivar	Shiraz BVRC12	Shiraz 1654	Sauvignon Blanc	Flame Seedless	Ruby Seedless
*d*_h2_ (μm)	12.8 ± 0.38*a*	11.4 ± 0.41*b*	11.8 ± 0.76*ab*	10.6 ± 0.25*b*	11.1 ± 0.29*b*
VD (n mm^–2^)	3273 ± 298*b*	3696 ± 155*b*	4567 ± 296*a*	5135 ± 236*a*	4920 ± 113*a*
*A*_lumen_ (%)	0.17 ± 0.02	0.15 ± 0.0093	0.16 ± 0.0092	0.15 ± 0.0022	0.17 ± 0.0019
*K* (kg m^–1^ MPa^–1^ s^–1^)	0.75 ± 0.097	0.63 ± 0.091	0.54 ± 0.080	0.44 ± 0.024	0.51 ± 0.020

*Data are presented as mean ± s.e.m. (n = 3 or 4). Where different lower-case letters are shown, it indicates differences between cultivars (Fisher’s LSD test, P < 0.05).*

**FIGURE 10:**
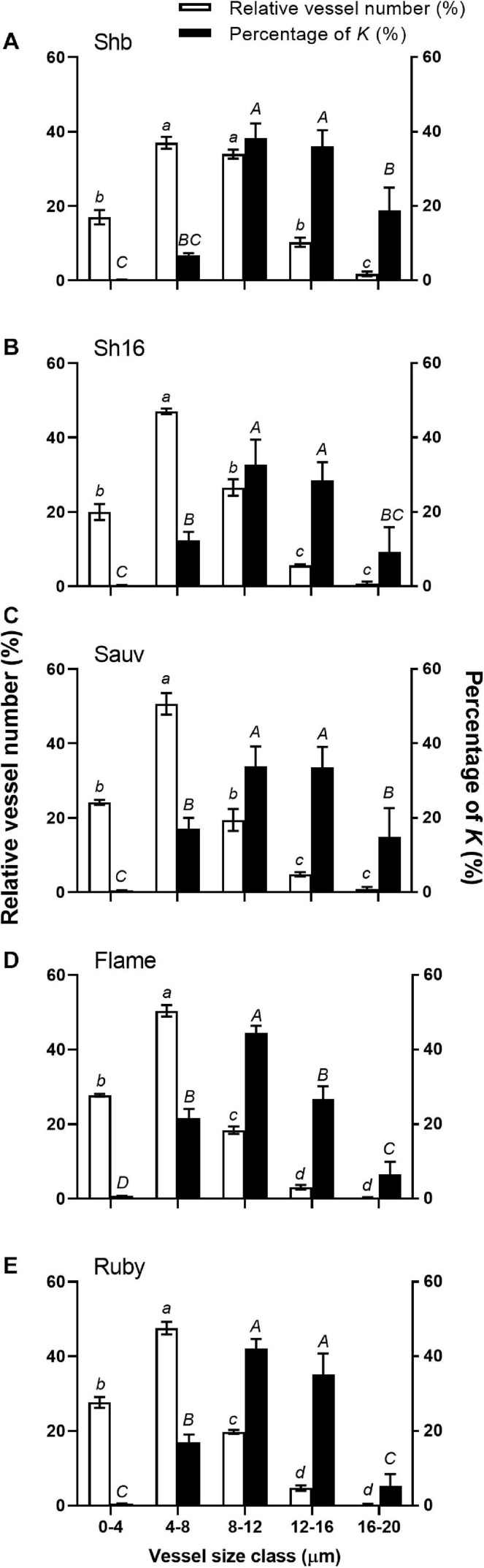
Relative abundance of vessels in five diameter classes (white bars) and their relative contribution to the potential area-specific hydraulic conductivity (*K*, in percent, black bars) in the receptacle of Shiraz BVRC12 **(A)**, Shiraz 1654 **(B)**, Sauvignon Blanc **(C)**, Flame Seedless **(D)**, and Ruby Seedless **(E)**. Data are presented as mean ± s.e.m. (*n* = 3 or 4). Where lower-case letters are shown, it indicates differences between diameter classes in the abundance of vessels and where upper-case letters are shown, it indicates differences between the relative contributions to *K* (Fisher’s LSD test, *P* < 0.05).

When vessels were divided into five diameter size classes (i.e., 4 μm interval), two predominant vessel classes were apparent in Shiraz BVRC (4–8 and 8–12 μm), while in Shiraz 1654, Sauvignon Blanc, Flame Seedless and Ruby Seedless, approximately 60% of the vessels were found to be smaller than 8 μm (Figure 10). The total xylem area-specific hydraulic conductivity (*K*) derived from the Hagen–Poiseuille equation demonstrated that a significant proportion of *K* could be attributed to xylem vessels in both the 8–12 and 12–16 μm diameter classes in Shiraz BVRC12, Shiraz 1654, Sauvignon Blanc and Ruby Seedless (Figures 10A–C,E). However, in Flame Seedless, the 8–12 μm xylem size class contributed more to *K* than the other size classes (Figure 10D).

**FIGURE 11:**
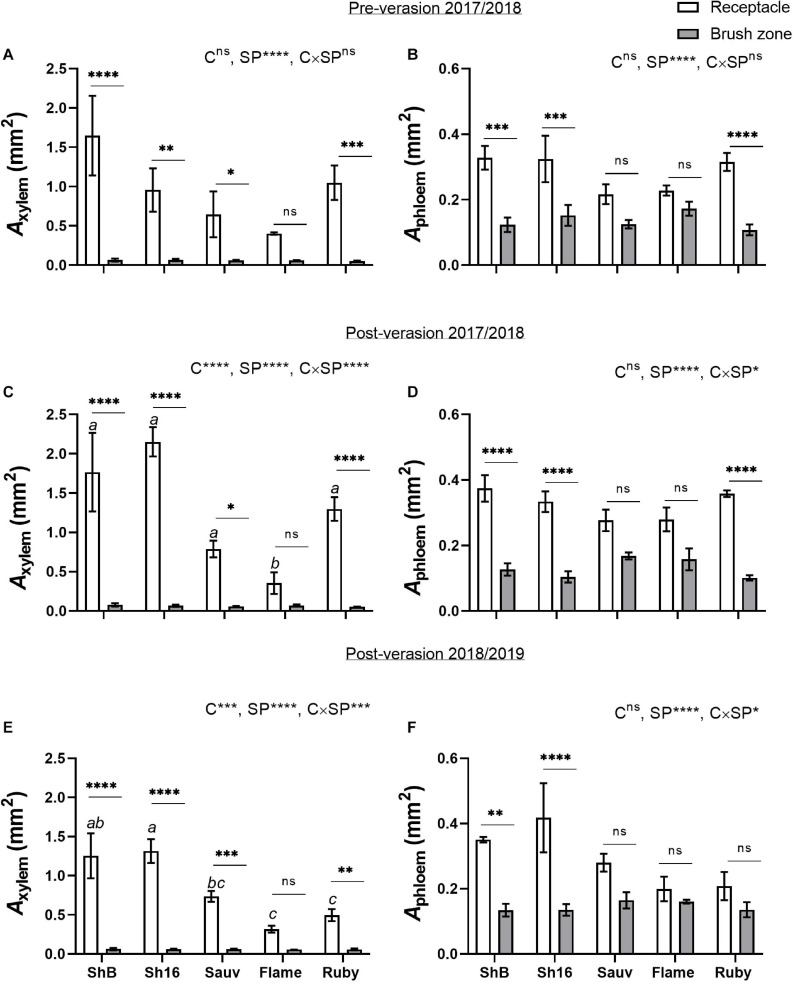
Cross-sectional areas of xylem (*A*_xylem_) **(A,C,E)** and phloem (*A*_phloem_) **(B,D,F)** in receptacles and brush zones of Shiraz BVRC12 (ShB), Shiraz 1654 (Sh16), Sauvignon Blanc (Sauv), Flame Seedless (Flame), and Ruby Seedless (Ruby) before and after veraison in the 2017/2018 season and after veraison in the 2018/2019 season (data of brush zone 2 was used for Post-veraison 2018/2019). Data are presented as mean ± s.e.m. (*n* = 3 or 4). The ANOVA results are given for cultivar (C), section position (SP) and cultivar × section positon (C × SP) interaction. Levels of significance are *P < 0.05, **P < 0.01, ***P < 0.001, ****P < 0.0001 and ^ns^, not significant. Where different lower-case letters are shown, it indicates differences in *A*_xylem_ between cultivars and there is no difference in *A*_phloem_ between cultivars in the receptacle after veraison in both season (Fisher’s LSD test, *P* < 0.05).

**FIGURE 12:**
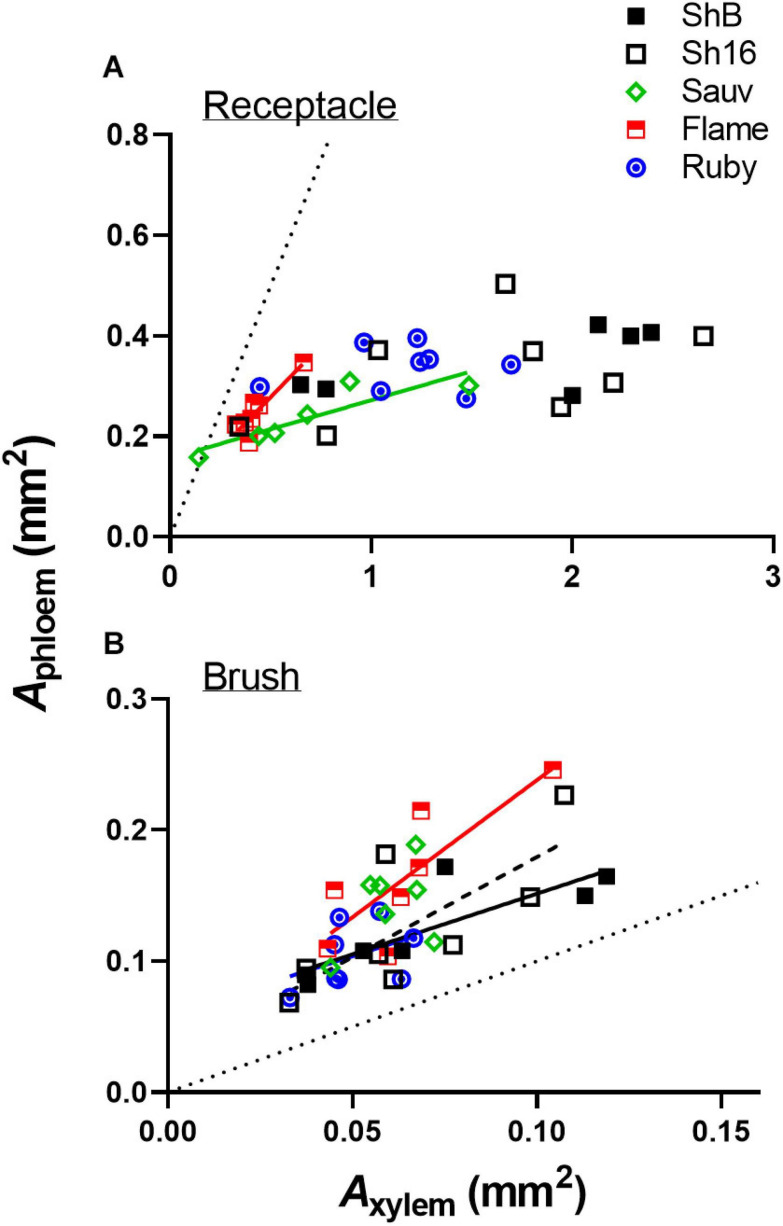
Correlations between *A*_phloem_ and *A*_xylem_ in receptacle **(A)** and brush zone **(B)** for pre- and post-veraison Shiraz BVRC12 (ShB), Shiraz 1654 (Sh16), Sauvignon Blanc (Sauv), Flame Seedless (Flame), and Ruby Seedless (Ruby) in 2017/2018 season. Where linear lines are shown, this indicates a positive correlation (Pearson’s *r*, *P* < 0.05) between *A*_phloem_ and *A*_xylem_ in the respective cultivar. Dotted diagonal lines are y = x.

The total cross-sectional areas of xylem (*A*_*xylem*_) and phloem (*A*_phloem_) in the receptacle and brush zone were estimated (Figure 11) and it was evident that in the two Shiraz clones after veraison, both *A*_*xylem*_ and *A*_phloem_ were larger in the receptacle than in the brush zone (Figures 11C–F). *A*_xylem_ in the receptacle was different amongst cultivars but not in the brush zone after veraison (Figures 11C,E). The two Shiraz clones exhibited larger *A*_xylem_ post-veraison relative to Flame Seedless in both seasons and to Ruby Seedless in the second season (Figures 11C,E). There was no difference in *A*_phloem_ between cultivars in either the receptacle or the brush zone (Figures 11B,D,F). A relatively small region of phloem surrounded the xylem in the receptacle (Figures 4, 5, 11) and noticeably *A*_xylem_ of both the Shiraz clones was approximately five times larger than *A*_phloem_ (Figure 11). In the brush zone, *A*_phloem_ was approximately twice as large as *A*_xylem_ for all cultivars (Figure 11). In individual samples before and after veraison in the 2017/2018 season, the *A*_phloem_/*A*_xylem_ ratio across all cultivars was below 1 for nearly all receptacles (Figure 12A). *A*_phloem_ was positively correlated with *A*_xylem_ in the receptacles of Sauvignon Blanc (Pearson’s *r* = 0.89, *P* = 0.0165) and in Flame Seedless (Pearson’s *r* = 0.90, *P* = 0.0064) (Figure 12A). In the brush zone the *A*_phloem_/*A*_xylem_ ratio was above 1 across all cultivars (Figure 12B). *A*_phloem_ was positively correlated with *A*_xylem_ in Shiraz BVRC12 (Pearson’s *r* = 0.84, *P* = 0.0174), Shiraz 1654 (Pearson’s *r* = 0.76, *P* = 0.0301) and Flame Seedless (Pearson’s *r* = 0.81, *P* = 0.0258) (Figure 12B).

## Discussion

A clearer understanding of the berry’s vascular anatomy is required for a more accurate description of berry vascular conductance and the ultimate impact on fruit quality. This study has characterized the vascular structure from the receptacle through to the grape berry brush zone in five grape cultivars, three seeded wine grapes and two seedless table grapes, prior to and after the onset of ripening. A consistent feature observed in all cultivars and sampling times was the change in vascular arrangement from the pedicel into the grape brush zone. In the receptacle, the vascular bundles were arranged so that the centrally located xylem was internal to and surrounded by the phloem, an arrangement common to other fruit receptacles such as the raspberry (Williamson et al., 1994). At the junction of the grape receptacle and the berry, this organization altered, such that the solid core of secondary xylem became subdivided into smaller, separate bundles, each of which was surrounded by phloem (amphicribral-like arrangement). Finally, in the brush zone the vascular tissues were again arranged into separate collateral bundles, however, with the phloem now orientated internal to the xylem. This was already apparent in the early pre-veraison stage (Figure 4 and Supplementary Figure 5). The change in vascular arrangement between plant organs is not delimited to the grape. In *Leea* plants, an allied genus of *Vitis*, the central bundles also become inverted before they enter the carpels, with the phloem on the adaxial (inner) side of the vascular bundles (Rafei, 1941). Since a grape berry originates from two fused carpels (Ağaoğlu, 1971; Considine and Knox, 1979) this change in the vascular pattern suggests high morphological plasticity which could facilitate vascular connectivity between the pedicel and the berry (Endress, 2019). The inversion in vascular arrangement described here within the grape berry may have been instigated by spatial differences in the concentration of hormones, including auxin, cytokinin, and gibberellin, all playing a role in cambium cell proliferation and fate through gene-expression regulation (Loomis and Torrey, 1964; Uggla et al., 1996; Tuominen et al., 1997; Ye, 2002; Israelsson et al., 2005; Nieminen et al., 2008). We may hypothesize, that the temporary spatial separation between the xylem and phloem would impair the transfer of substances between the two systems, and their reunion would reinstate this.

In contrast to the central vasculature, the peripheral vascular bundles exhibited the more common collateral arrangement (Figures 6, 8), as seen previously in Chardonnay (Chatelet et al., 2008a). The inverted phloem in the brush zone vascular system is likely to be a by-product of carpel fusion. However, whether there is functional implication remains to be determined. Here two suggestions are offered as preliminary avenues for further research. The first proposition is that the inversion facilitates the isolation and retrieval of leaked solutes from the phloem cells. Typically, the sieve element-companion cell complex is embedded in phloem parenchyma cells. Radial exchange of phloem contents between these parenchyma cells ensures minimal loss and efficient transport to the sink (Thorpe et al., 2005), and may be facilitated with transfer cells (Andriunas et al., 2013). The orientation of the phloem tissues toward each other in the central axis may maximize the retrieval of sucrose and other metabolites and help uphold the hydrostatic pressure of the sieve tubes and thus maintain the flow to the seeds and the distal portion of the berry. The second potential explanation addresses the hypoxic conditions within the center of the berry. The grape mesocarp has been observed to become hypoxic during berry ripening (Xiao et al., 2018a, b). The central locular cavity and surrounding tissues, as well as the layers of cells underneath the berry skin require oxygen to sustain living cell function during berry ripening and the metabolic activity of phloem unloading. Because phloem consists of living cells (Van Bel, 2003), the inward growth of phloem within the brush zone central vascular bundles and the outward growth of peripheral phloem may provide better access to oxygen for maintaining cell viability and the active transport required for phloem (un)loading (De Schepper et al., 2013). Higher oxygen concentrations have been measured within the central locular region of the berry and this may be the result of a direct pathway from the lenticels located on the pedicel (Xiao et al., 2018b). Further research will be required to substantiate the role of oxygen access in vascular orientation.

Phenolic compounds have been localized in the Muscat Gordo Blanco berry brush and skin in comparable concentration at 10 and 17°Brix ripeness (Coombe, 1987). The methanol fixation is likely to have eliminated the possible chloroplast fluorescence from tissue surrounding the brush zone central vascular bundles, enhancing the strong green fluorescence from the phenolic compounds in the cell lumina (Figure 7; Donaldson and Williams, 2018). However, methanol fixation resulted in relatively stronger cell-wall fluorescence only in the brush zone of Shiraz (Figures 4, 7 and Supplementary Figure 5).

The reduction in the size of xylem (*d*_*h*__1_), and total tissue area (*A*_xylem_ and *A*_phloem_) from the receptacle into the grape brush zone varied between cultivars. Conducting xylem vessels taper to compensate for the lengthy water transport route from the ground upwards along their length (West et al., 1999). The mean hydraulically weighted xylem diameter previously found in the rachises of Shiraz and Grenache, ranged between approximately 21.05 and 25.41 μm (Scharwies and Tyerman, 2017), was larger than that of the berry/pedicel components (Figure 9). Within the grapevine shoot, vessel diameter reduced from the basal to distal region and was associated with a reduction in hydraulic conductivity (Lovisolo and Schubert, 1998). The reduction of area specific conductivity due to the decrease of xylem vessel size is consistent with Poiseuille’s law (Tyree and Ewers, 1991). Indeed, hydraulic conductance differed within the individual berry/pedicel components; both proximal and distal portions of the berry were characterized with lower conductivity than the receptacles in Shiraz and Chardonnay (Tyerman et al., 2004). The tapering xylem vessels from the receptacle into the berry junction and brush contribute to the larger hydraulic resistance in the berry portion relative to the receptacle (Figure 9).

Heterogeneity in receptacle-related xylem anatomical and calculated hydraulic parameters were observed between cultivars. Larger xylem vessels (*d*_2_ ≥ 8 μm), abundant in the Shiraz receptacle, can potentially have a large contribution to the total calculated *K*, according to the Hagen-Poiseuille equation. This could aid excessive xylem back-flow in Shiraz during late-ripening and lead to berry shrivel (Tilbrook and Tyerman, 2009). Using a root pressure probe it was demonstrated that Shiraz had greater flow out of the berry relative to Chardonnay and it could explain an approximate loss of 7% of the berry volume per day. In the receptacle, the derived total xylem area-specific hydraulic conductivity did not differ between cultivars (Table 1), but it is likely the receptacle could function differently under stress as a result of the heterogeneity in vessel size and density as observed here. There vessel characteristics would have implications on cavitation resistance (Cai and Tyree, 2010) and transverse pressure gradients across vessel bundles (Bouda et al., 2019). The derived hydraulic conductivity (*K*) should be interpreted carefully because any errors in the measured diameter are amplified by the formula used to estimate the conduit conductivity (Eq. 3). Furthermore, hydraulic conductance capability does not depend on xylem size alone. The hydraulic conductivity in the receptacle of post-veraison (over 80 DAA) Cabernet Sauvignon berries, normalized to the receptacle cross-sectional area, can be converted to approximately 2.5 × 10^–4^ kg m^–1^ MPa^–1^ s^–1^ (Knipfer et al., 2015). If the normalization was carried out using the xylem-specific area within the receptacle, an increase in the hydraulic conductivity can be expected, but our calculated *K* was still over 1,000-fold larger (Table 1). Other mechanisms such as inter-conduit connection observed in the berry pedicel (Knipfer et al., 2015) and pit membrane structure (Hajek et al., 2014) might be associated with the smaller measured *K*.

A positive correlation between *A*_phloem_ and *A*_xylem_ into and within the central bundles indicates proportional structural investment in phloem and xylem (Savage et al., 2016). The strength of the correlation varied across cultivars and in different parts of the berry (receptacle or brush zone), but the *A*_phloem_/*A*_xylem_ ratio of the central bundles in the brush zone was consistently larger than in the receptacle before and after veraison (Figure 12). This could mean: (1) due to differences in functional aspects of the berry and pedicel, phloem size increased in the berry brush zone portion to compensate for the largely reduced xylem conductance so that overall water influx is maintained during ripening (Hölttä et al., 2009); and/or (2) the procambial cell identity was established before mass sugar influx occurred post-veraison. The poor correlation between total soluble solids with *A*_phloem_ suggests that other parameters such as phloem sucrose concentration and flux rates are also important drivers for this quality parameter (Zhu et al., 2018). Sugar transporter genes are upregulated at veraison as are sugar metabolism enzymes (Deluc et al., 2007; Hayes et al., 2007) and may play a stronger role in establishing flux rates than *A*_phloem_. Similarly, berry growth is sensitive to surface transpiration and this is dependent on cuticle properties (Zhang and Keller, 2017) and environmental conditions such as vapor pressure (Zhu et al., 2018).

## Conclusion

The vascular connection between the pedicel and the grape berry has been characterized in commercial grape cultivars. Xylem anatomical and derived hydraulic parameters as well as total vascular tissue area were found to vary between cultivars and in receptacle/berry components, which could result in different *K* between cultivars and affect water retention of the berries. *A*_phloem_/*A*_xylem_ ratio was consistently larger in the berry brush zone than in the receptacle regardless of seededness. Phloem size was consistent between cultivars in both the pedicel and brush zone. This suggests other cultivar dependent growth regulators such as loading rate and phloem sucrose concentrations could play a more important role in dictating berry size and sugar accumulation. All cultivars exhibited an inversion in the vascular arrangement within the central axis of the berry as it traversed from the receptacle into the brush zone. It is possible that this inversion has implications on phloem vitality, the development of the seeds and the central pericarp tissues and in the exchange of solutes and water between xylem and phloem.

## Data Availability Statement

The original contributions presented in the study are included in the article/Supplementary Material, further inquiries can be directed to the corresponding author/s.

## Author Contributions

ZX, SC, RW, AG, and SR organized the database. ZX, SC, RW, ST, and SR carried out the image analysis. ZX, ST, and SR carried out the statistical analysis. ZX wrote the first draft of the manuscript. All authors contributed to manuscript revision, read, and approved the submitted version, contributed to conception, and design of the study.

## Conflict of Interest

SC was employed by company Noble Research Institute LLC. The remaining authors declare that the research was conducted in the absence of any commercial or financial relationships that could be construed as a potential conflict of interest.

## Supplementary Material

Supplementary Figure 1 Example images of xylem in receptacle **(A,D)**, receptacle/berry junction **(B,E)** and brush zone 2 **(C,F)** in post-veraison Shiraz BVRC12 and 1654 in 2018/2019 season. The white elliptic selections mark xylem vessels that were chosen randomly. Scale bars are 50 μm.

Supplementary Figure 2 Example of a cross-section of Sauvignon Blanc berry receptacle illustrating consecutive steps of the automatic xylem selection protocol. A region of the xylem is cropped and binarized before the individual vessels are selected and measured. Scales bars are 100 μm.

Supplementary Figure 3 Example images of hand tracings to measure the area of phloem (red line) and xylem (yellow line) in a Shiraz 1654 post-veraison receptacle **(A)** and brush zone 2 **(B)**. Scale bars are 100 μm.

Supplementary Figure 4 Berry weight of Shiraz BVRC12 (ShB) and 1654 (Sh16), Sauvignon Blanc (Sauv), Flame Seedless (Flame), and Ruby Seedless (Ruby) during season 2017/2018. Each point represents the average of 10 berries.

Supplementary Figure 5 Sections of receptacles **(A,D,G,J,M,P)**, receptacle/berry junction **(B,E,H,K,N,Q)**, brush zone (2–4 mm from junction) **(C,F,I,L,O,R)**, stained with acridine orange and imaged under UV, of methanol stored Shiraz 1654 **(A,B,C,J,K,L)**, Sauvignon Blanc **(D,E,F,M,N,O)**, and Ruby Seedless **(G,H,I,P,Q,R)** at around 44 (pre-veraison) and 85 (post-veraison) days after anthesis (DAA) in the 2017/2018 season. Bright blue indicates lignin (xylem and phloem fiber), pinkish orange indicates the location of phloem (x, xylem; p, phloem; r, xylem ray; f, phloem fiber). Excitation filter 330–385 nm, dichroic mirror 400 nm, barrier filter 420 nm. Scale bar = 100 μm.

Supplementary Figure 6 Sections of receptacles **(A,E,I)**, receptacle/berry junction **(B,F,J)**, brush zone 1 (2–4 mm from junction) **(C,G,K)**, brush zone 2 (4–6 mm from junction) **(D,H,L)**, stained with aniline blue fluorochrome and imaged under UV, of fresh berries of Shiraz 1654 **(A–D)**, Sauvignon Blanc **(E–H)**, and Ruby Seedless **(I–L)** at around 65 days after anthesis (post-veraison), in the 2018/2019 season. Blue color indicates lignin (xylem and phloem fiber). Bright fluorescent greenish blue indicates callose formed within the sections and primarily within phloem, red tint in images indicates chlorophyll (x, xylem; p, phloem; r, xylem ray; f, phloem fiber). Excitation filter 330–385 nm, dichroic mirror 400 nm, barrier filter 420 nm. Scale bar = 100 μm.
