# Supplementary figures and images for: Vascular Connections Into the Grape Berry: The Link of Structural Investment to Seededness
Source: Front Plant Sci. 2021 Apr 15;12:662433. doi: 10.3389/fpls.2021.662433 (PMC8083876; doi:10.3389/fpls.2021.662433)

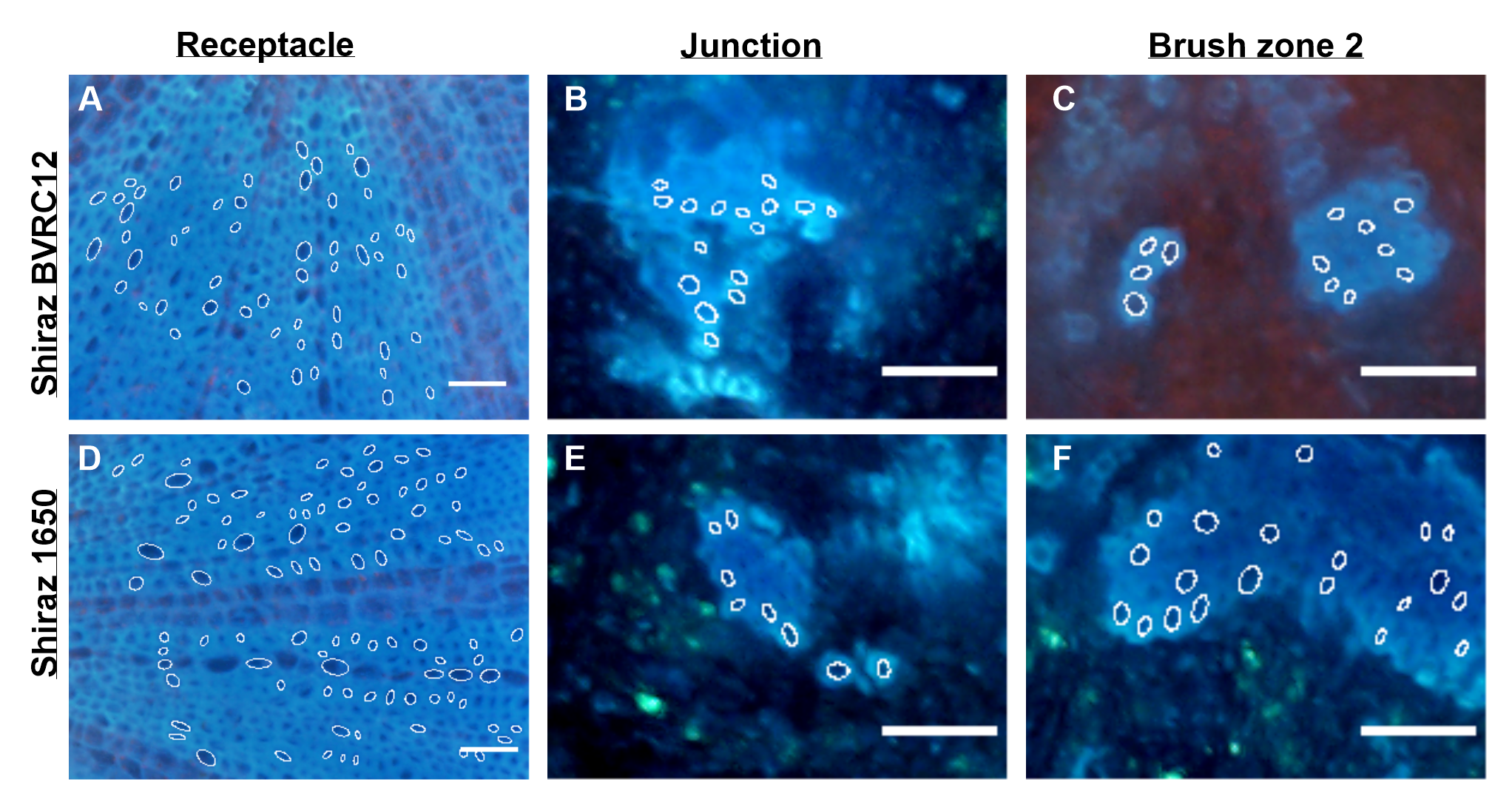

Supplement: Supplementary Figure 1 — Example images of xylem in receptacle (A,D), receptacle/berry junction (B,E) and brush zone 2 (C,F) in post-veraison Shiraz BVRC12 and 1654 in 2018/2019 season. The white elliptic selections mark xylem vessels that were chosen randomly. Scale bars are 50 μm. [file Image_1.TIF]

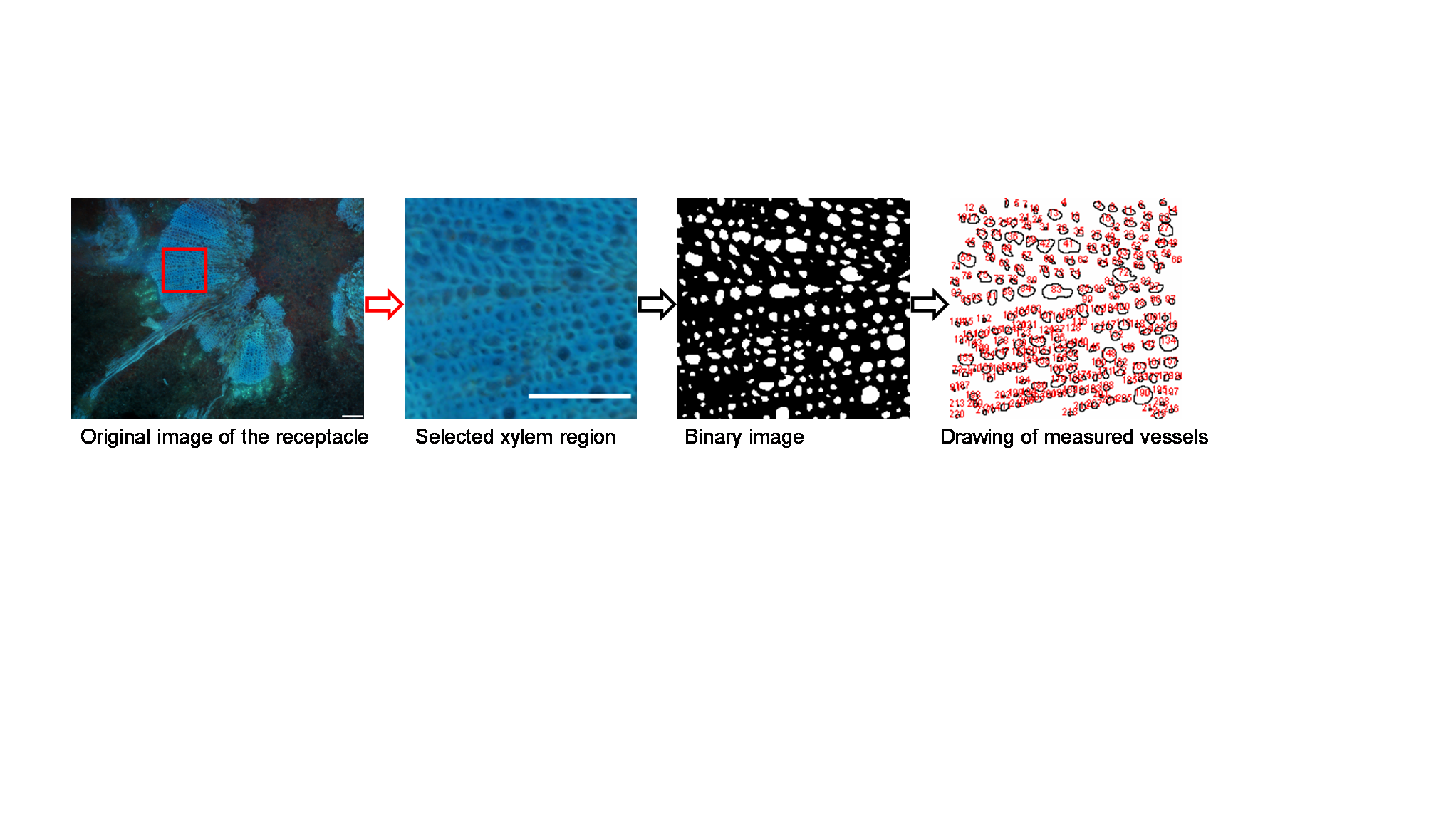

Supplement: Supplementary Figure 2 — Example of a cross-section of Sauvignon Blanc berry receptacle illustrating consecutive steps of the automatic xylem selection protocol. A region of the xylem is cropped and binarized before the individual vessels are selected and measured. Scales bars are 100 μm. [file Image_2.TIF]

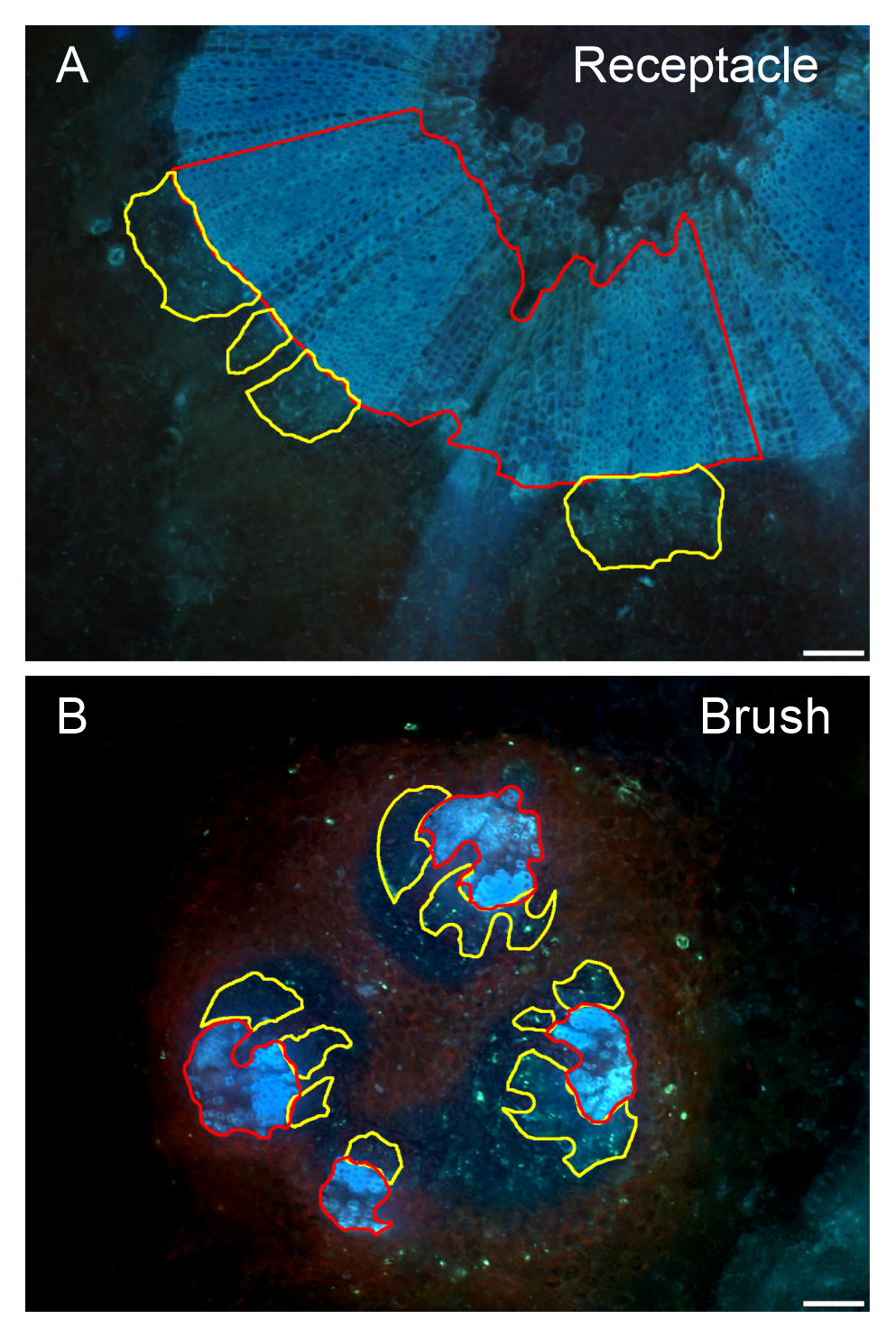

Supplement: Supplementary Figure 3 — Example images of hand tracings to measure the area of phloem (red line) and xylem (yellow line) in a Shiraz 1654 post-veraison receptacle (A) and brush zone 2 (B). Scale bars are 100 μm. [file Image_3.TIF]

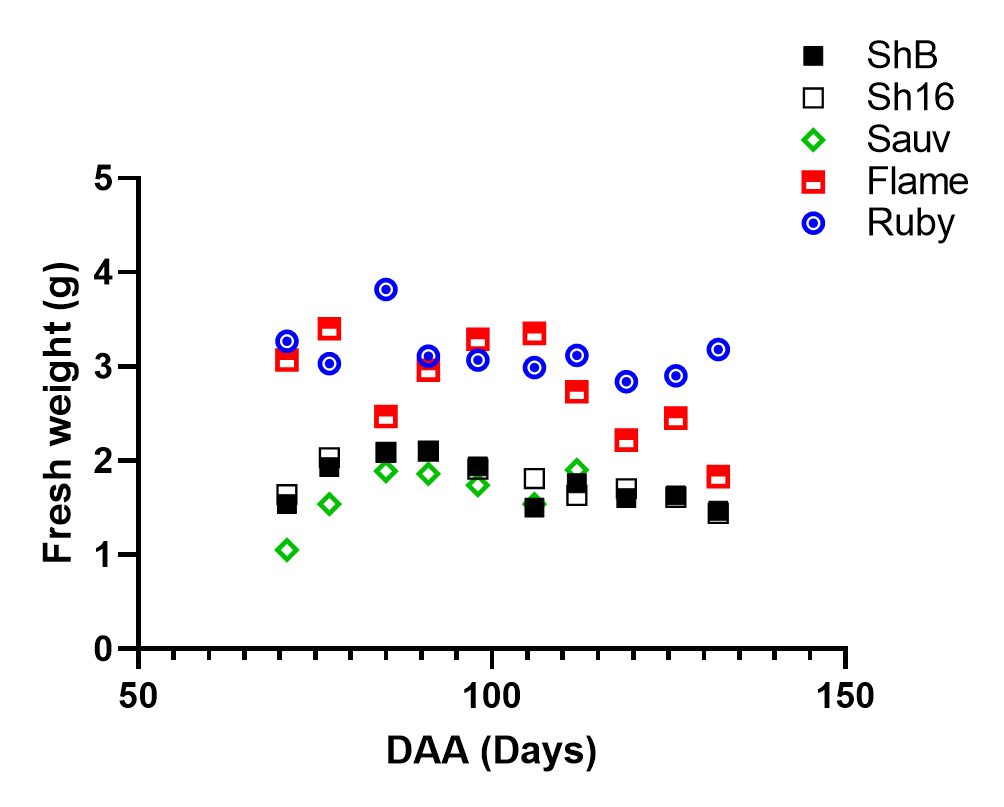

Supplement: Supplementary Figure 4 — Berry weight of Shiraz BVRC12 (ShB) and 1654 (Sh16), Sauvignon Blanc (Sauv), Flame Seedless (Flame), and Ruby Seedless (Ruby) during season 2017/2018. Each point represents the average of 10 berries. [file Image_4.TIF]

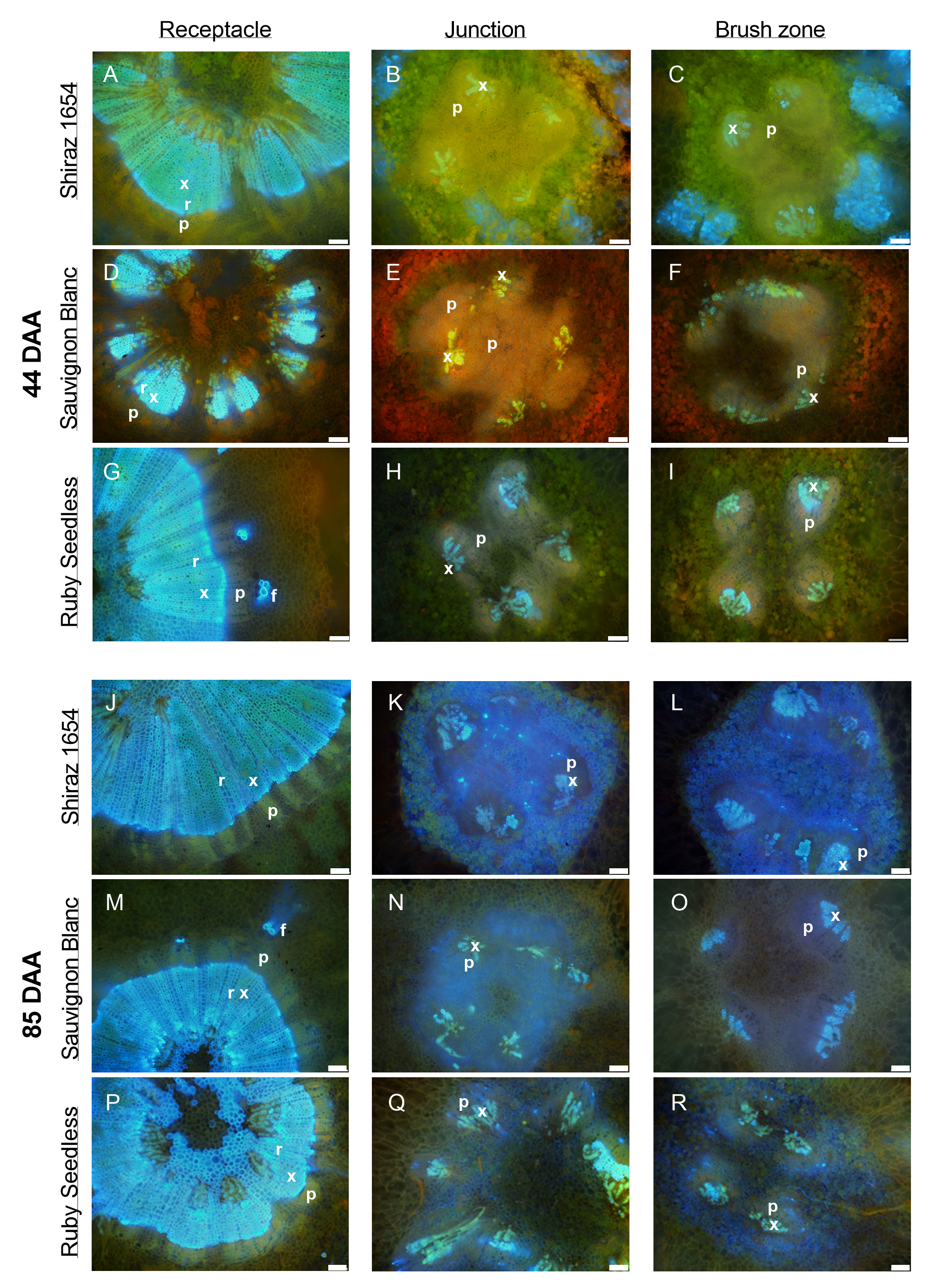

Supplement: Supplementary Figure 5 — Sections of receptacles (A,D,G,J,M,P), receptacle/berry junction (B,E,H,K,N,Q), brush zone (2–4 mm from junction) (C,F,I,L,O,R), stained with acridine orange and imaged under UV, of methanol stored Shiraz 1654 (A,B,C,J,K,L), Sauvignon Blanc (D,E,F,M,N,O), and Ruby Seedless (G,H,I,P,Q,R) at around 44 (pre-veraison) and 85 (post-veraison) days after anthesis (DAA) in the 2017/2018 season. Bright blue indicates lignin (xylem and phloem fiber), pinkish orange indicates the location of phloem (x, xylem; p, phloem; r, xylem ray; f, phloem fiber). Excitation filter 330–385 nm, dichroic mirror 400 nm, barrier filter 420 nm. Scale bar = 100 μm. [file Image_5.TIF]

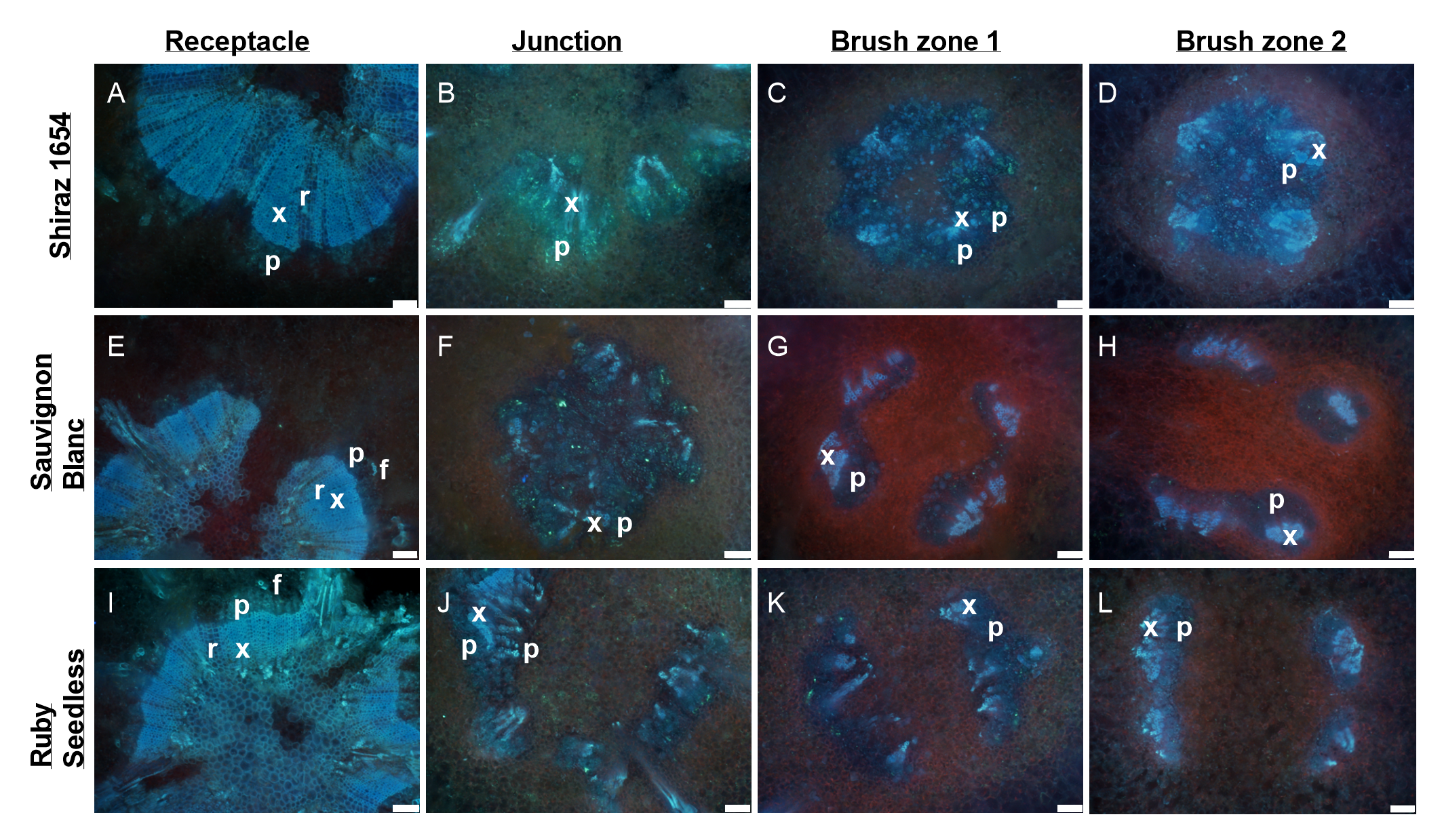

Supplement: Supplementary Figure 6 — Sections of receptacles (A,E,I), receptacle/berry junction (B,F,J), brush zone 1 (2–4 mm from junction) (C,G,K), brush zone 2 (4–6 mm from junction) (D,H,L), stained with aniline blue fluorochrome and imaged under UV, of fresh berries of Shiraz 1654 (A–D), Sauvignon Blanc (E–H), and Ruby Seedless (I–L) at around 65 days after anthesis (post-veraison), in the 2018/2019 season. Blue color indicates lignin (xylem and phloem fiber). Bright fluorescent greenish blue indicates callose formed within the sections and primarily within phloem, red tint in images indicates chlorophyll (x, xylem; p, phloem; r, xylem ray; f, phloem fiber). Excitation filter 330–385 nm, dichroic mirror 400 nm, barrier filter 420 nm. Scale bar = 100 μm. [file Image_6.TIF]
